# Supplementary material for: Post-Surgical Depositions of Blood Products Are No Major Confounder for the Diagnostic and Prognostic Performance of CEST MRI in Patients with Glioma
Source: Biomedicines. 2023 Aug 23;11(9):2348. doi: 10.3390/biomedicines11092348 (PMC10525358; doi:10.3390/biomedicines11092348)
Supplement: Supplementary file 1 [file biomedicines-11-02348-s001.zip › Supplements_final.pptx]

## Slide 1
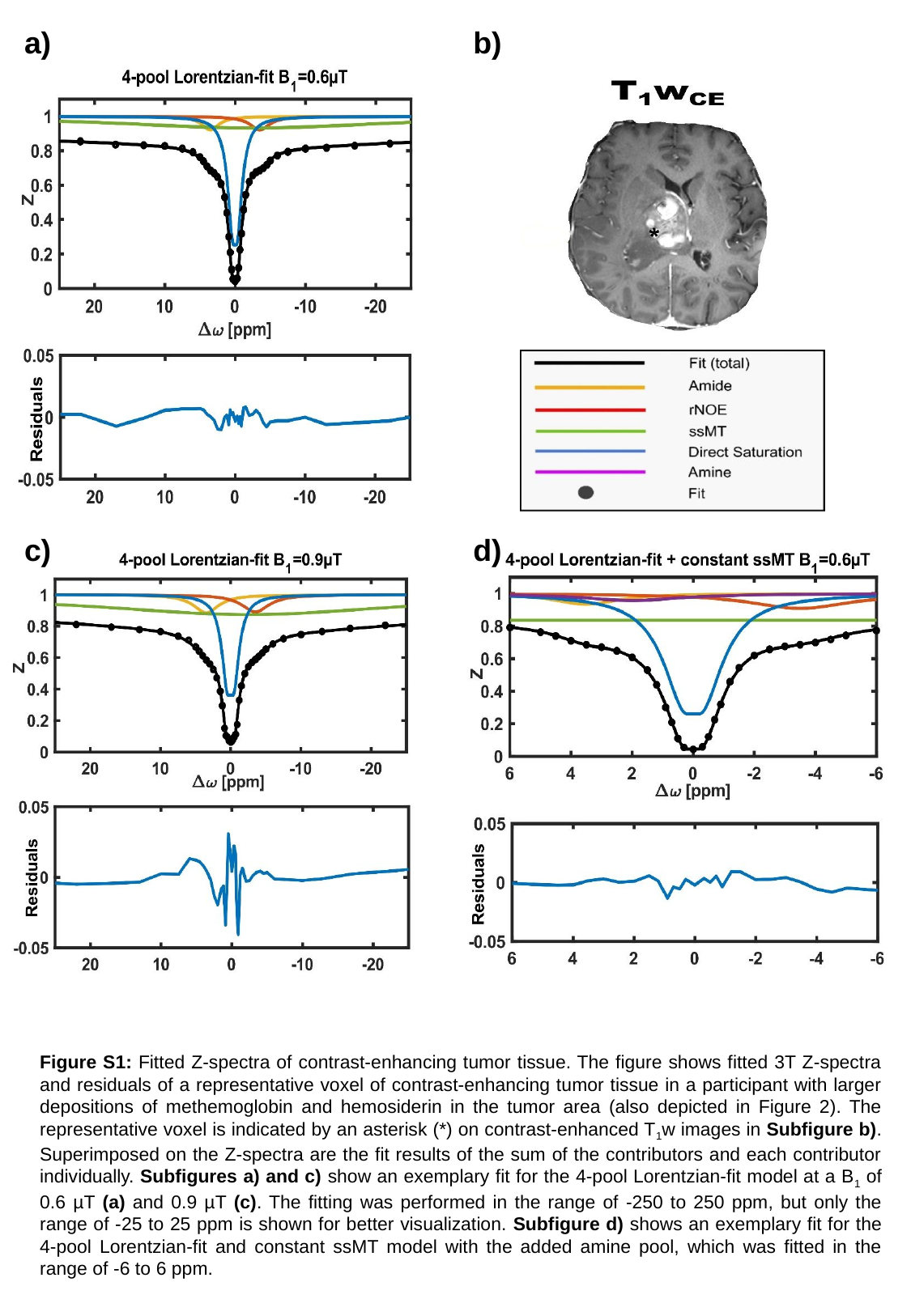

a)
b)
c)
d)
Figure S1: Fitted Z-spectra of contrast-enhancing tumor tissue. The figure shows fitted 3T Z-spectra and residuals of a representative voxel of contrast-enhancing tumor tissue in a participant with larger depositions of methemoglobin and hemosiderin in the tumor area (also depicted in Figure 2). The representative voxel is indicated by an asterisk (*) on contrast-enhanced T1w images in Subfigure b). Superimposed on the Z-spectra are the fit results of the sum of the contributors and each contributor individually. Subfigures a) and c) show an exemplary fit for the 4-pool Lorentzian-fit model at a B1 of 0.6 µT (a) and 0.9 µT (c). The fitting was performed in the range of -250 to 250 ppm, but only the range of -25 to 25 ppm is shown for better visualization. Subfigure d) shows an exemplary fit for the 4-pool Lorentzian-fit and constant ssMT model with the added amine pool, which was fitted in the range of -6 to 6 ppm.

## Slide 2
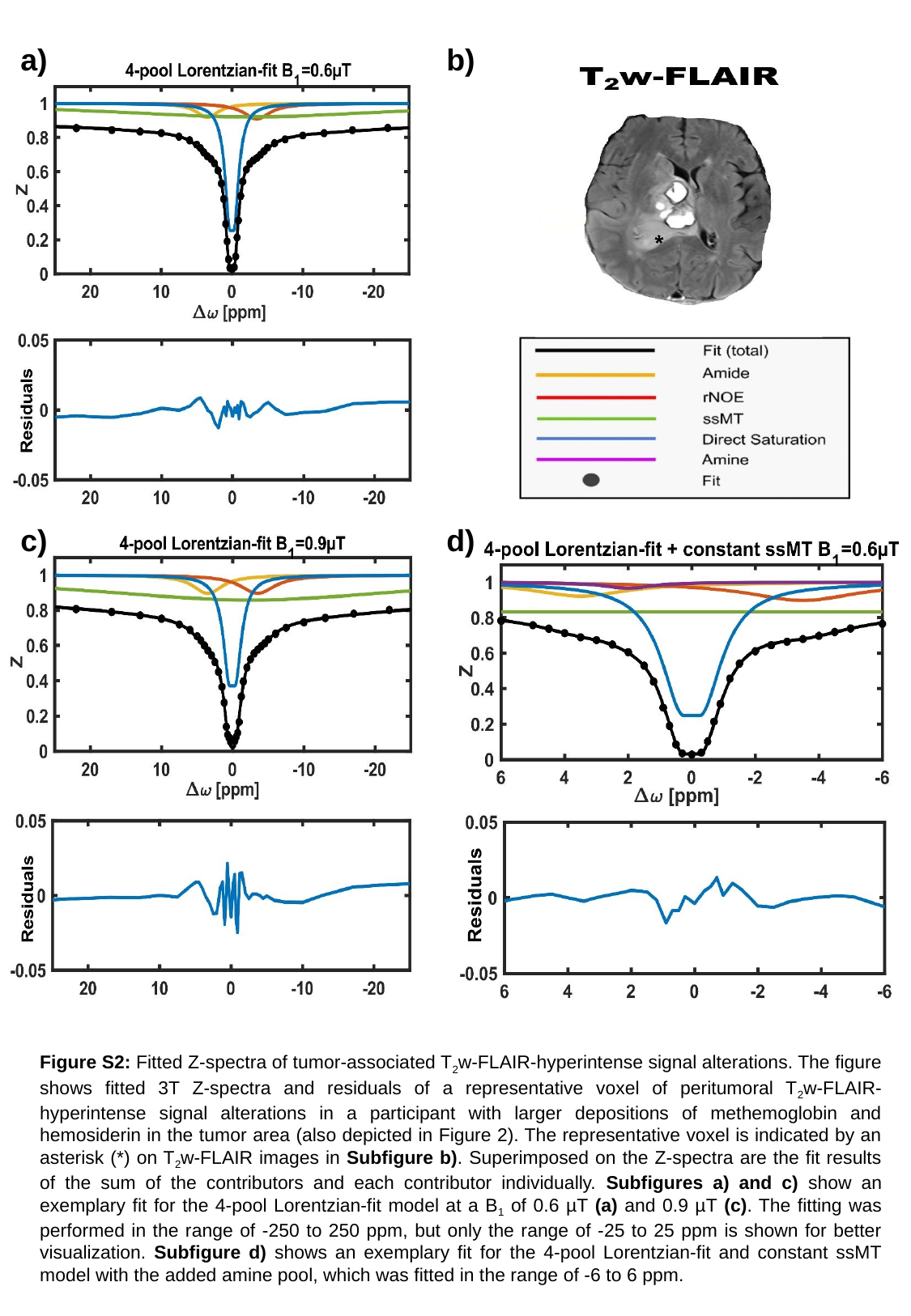

a)
b)
c)
d)
Figure S2: Fitted Z-spectra of tumor-associated T2w-FLAIR-hyperintense signal alterations. The figure shows fitted 3T Z-spectra and residuals of a representative voxel of peritumoral T2w-FLAIR-hyperintense signal alterations in a participant with larger depositions of methemoglobin and hemosiderin in the tumor area (also depicted in Figure 2). The representative voxel is indicated by an asterisk (*) on T2w-FLAIR images in Subfigure b). Superimposed on the Z-spectra are the fit results of the sum of the contributors and each contributor individually. Subfigures a) and c) show an exemplary fit for the 4-pool Lorentzian-fit model at a B1 of 0.6 µT (a) and 0.9 µT (c). The fitting was performed in the range of -250 to 250 ppm, but only the range of -25 to 25 ppm is shown for better visualization. Subfigure d) shows an exemplary fit for the 4-pool Lorentzian-fit and constant ssMT model with the added amine pool, which was fitted in the range of -6 to 6 ppm.

## Slide 3
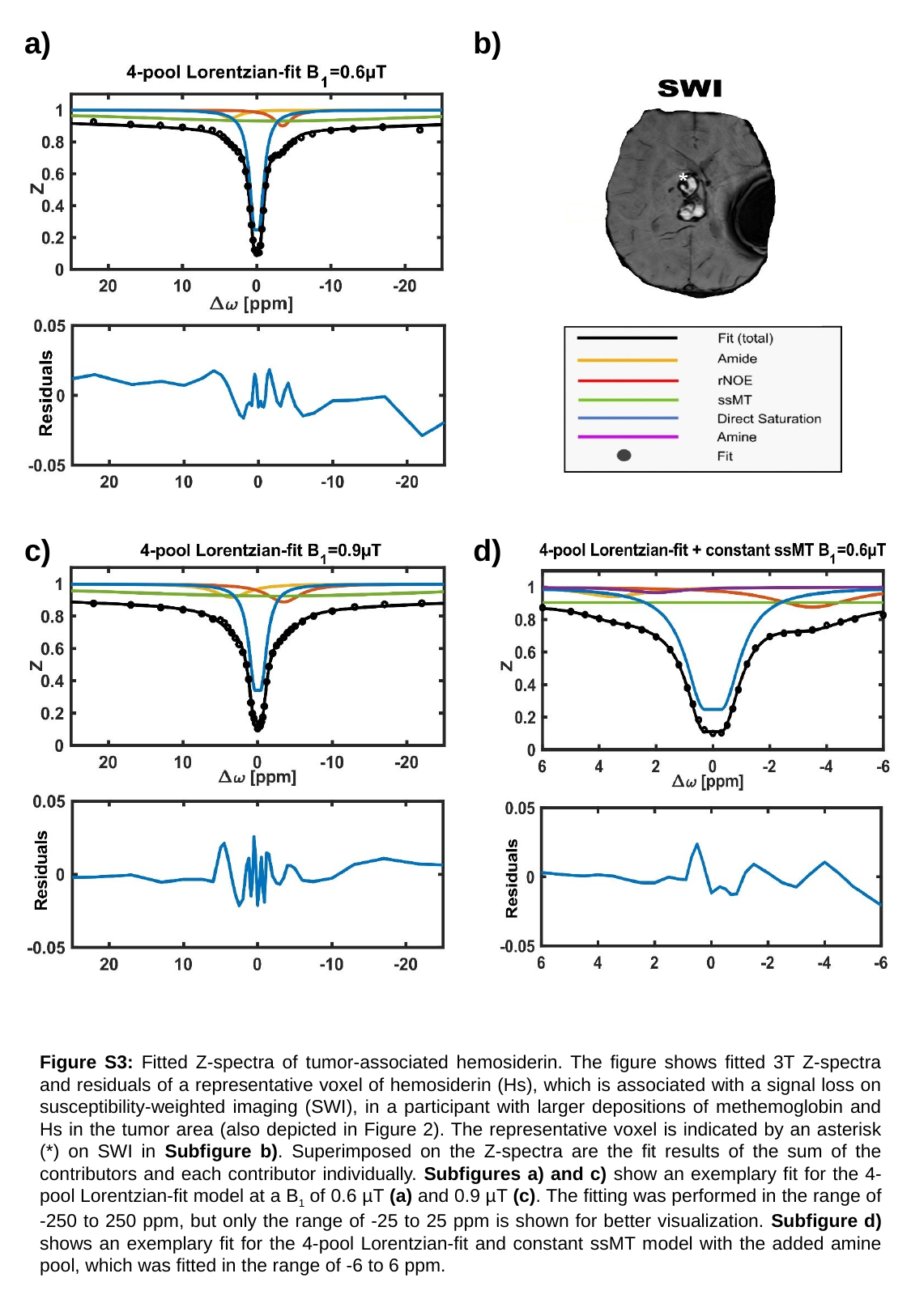

a)
b)
c)
d)
Figure S3: Fitted Z-spectra of tumor-associated hemosiderin. The figure shows fitted 3T Z-spectra and residuals of a representative voxel of hemosiderin (Hs), which is associated with a signal loss on susceptibility-weighted imaging (SWI), in a participant with larger depositions of methemoglobin and Hs in the tumor area (also depicted in Figure 2). The representative voxel is indicated by an asterisk (*) on SWI in Subfigure b). Superimposed on the Z-spectra are the fit results of the sum of the contributors and each contributor individually. Subfigures a) and c) show an exemplary fit for the 4-pool Lorentzian-fit model at a B1 of 0.6 µT (a) and 0.9 µT (c). The fitting was performed in the range of -250 to 250 ppm, but only the range of -25 to 25 ppm is shown for better visualization. Subfigure d) shows an exemplary fit for the 4-pool Lorentzian-fit and constant ssMT model with the added amine pool, which was fitted in the range of -6 to 6 ppm.

## Slide 4
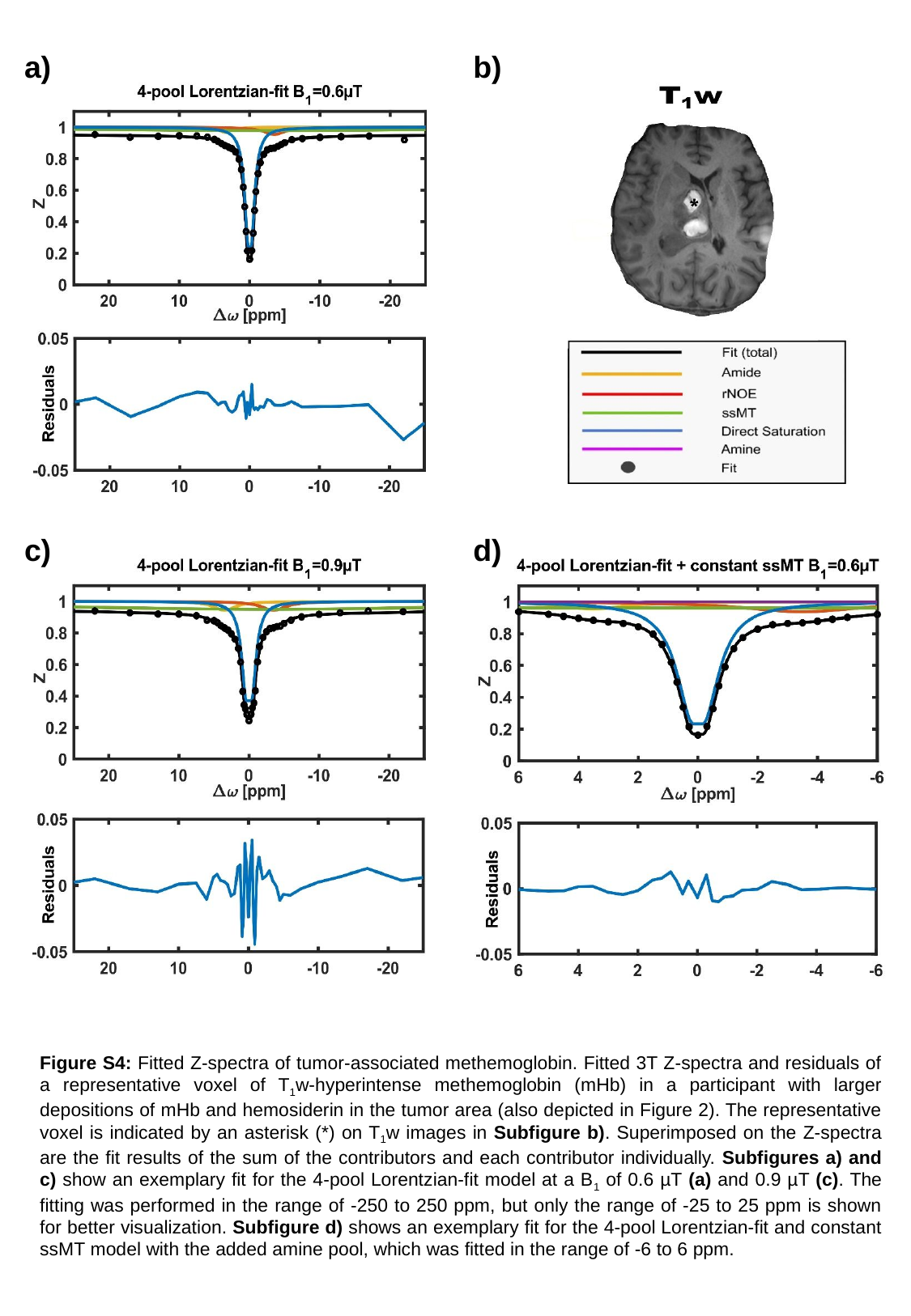

a)
b)
c)
d)
Figure S4: Fitted Z-spectra of tumor-associated methemoglobin. Fitted 3T Z-spectra and residuals of a representative voxel of T1w-hyperintense methemoglobin (mHb) in a participant with larger depositions of mHb and hemosiderin in the tumor area (also depicted in Figure 2). The representative voxel is indicated by an asterisk (*) on T1w images in Subfigure b). Superimposed on the Z-spectra are the fit results of the sum of the contributors and each contributor individually. Subfigures a) and c) show an exemplary fit for the 4-pool Lorentzian-fit model at a B1 of 0.6 µT (a) and 0.9 µT (c). The fitting was performed in the range of -250 to 250 ppm, but only the range of -25 to 25 ppm is shown for better visualization. Subfigure d) shows an exemplary fit for the 4-pool Lorentzian-fit and constant ssMT model with the added amine pool, which was fitted in the range of -6 to 6 ppm.
